# Protein surface functionalisation as a general strategy for facilitating biomimetic mineralisation of ZIF-8†
†Electronic supplementary information (ESI) available. See DOI: 10.1039/c8sc00825f


**DOI:** 10.1039/c8sc00825f

**Published:** 2018-03-09

**Authors:** Natasha K. Maddigan, Andrew Tarzia, David M. Huang, Christopher J. Sumby, Stephen G. Bell, Paolo Falcaro, Christian. J. Doonan

**Affiliations:** a Department of Chemistry and the Centre for Advanced Nanomaterials , The University of Adelaide , Adelaide , South Australia 5005 , Australia . Email: christian.doonan@adelaide.edu.au; b Institute of Physical and Theoretical Chemistry , Graz University of Technology , Stremayrgasse 9 , Graz 8010 , Austria

## Abstract

The durability of enzymes in harsh conditions can be enhanced by encapsulation within metal–organic frameworks (MOFs) *via* a process called biomimetic mineralisation. Herein we show that the surface charge and chemistry of a protein determines its ability to seed MOF growth. We demonstrate that chemical modification of amino acids on the protein surface is an effective method for systematically controlling biomimetic mineralisation by zeolitic imidazolate framework-8 (ZIF-8). Reaction of surface lysine residues with succinic (or acetic) anhydride facilitates biomimetic mineralisation by increasing the surface negative charge, whereas reaction of surface carboxylate moieties with ethylenediamine affords a more positively charged protein and hinders the process. Moreover, computational studies confirm that the surface electrostatic potential of a protein is a good indicator of its ability to induce biomimetic mineralisation. This study highlights the important role played by protein surface chemistry in encapsulation and outlines a general method for facilitating the biomimetic mineralisation of proteins.

## Abstract 2

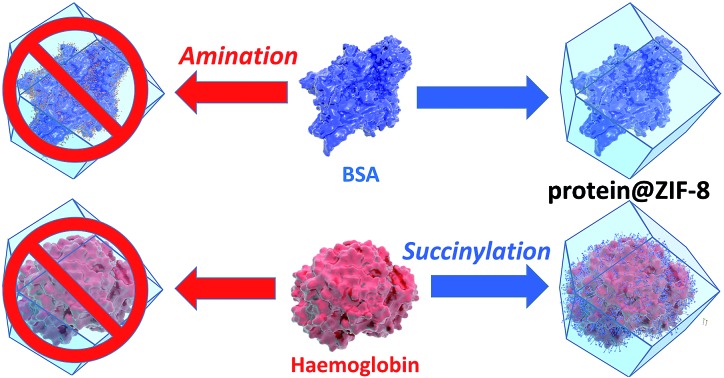
The surface charge and chemistry of a protein determines its ability to facilitate biomimetic mineralisation.

## Introduction

Metal–organic frameworks (MOFs) are a class of porous materials that are constructed from metal nodes connected *via* organic links.^1^ The chemical mutability of these building units offers broad scope for tailoring the properties of MOFs for specific applications such as gas separations, drug delivery and catalysis.^2^–^10^ A recent development in MOF chemistry is their use as matrices for encapsulating biomacromolecules, *e.g.* proteins and enzymes, *via* a one-pot synthetic approach termed ‘biomimetic mineralisation’.^11^–^16^ This strategy has also been extended to the synthesis of MOF-based biocomposites composed of viruses,^17^ and cells,^18^,^19^ and more recently to the co-encapsulation of gene-editing system CRISPR/CAS9.^20^ A salient feature of the MOF coating is that it can protect an encapsulated enzyme from inhospitable external environments (*e.g.* elevated temperatures or proteolytic media) while facilitating size-selective transport of substrates to the active site *via* its pore network.^21^–^23^ These properties are relevant to commercial bio-catalysis, for which strategies to improve enzyme durability are sought after.^24^

The most studied MOF for biomimetic mineralisation has been zeolitic imidazolate framework-8 (ZIF-8),^25^ a material of sodalite topology comprising tetrahedral Zn^2+^ ions connected *via* 2-methylimidazole (mIM) bridging units. ZIF-8 is porous (BET surface area *ca.* 1200 m^2^ g^–1^), stable in a wide range of organic solvents and can be synthesised in neat water.^26^,^27^ Standard conditions for the biomineralisation of ZIF-8 employ a stoichiometric ratio metal ions and organic linker (160 mM of mIM and 40 mM of metal salt) in an aqueous solution of 2 mg of protein at room temperature.^21^ While the presence of a biomacromolecule may enhance the kinetics, there are cases in which biomineralisation requires a higher excess of organic linker (mIM), or longer times, to engender ZIF formation.^28^ In order to maximise the efficacy and versatility of this promising strategy for the protection of biomacromolecules, a general approach is desirable.

A detailed understanding of the chemistry at the interface of the MOF and the biomacromolecule is necessary to develop this burgeoning area. A first step towards this aim is to ascertain how the surface chemistry of the protein influences the biomineralisation process. Preliminary data showed that MOF crystallisation was facilitated by the capacity of the biomacromolecule to attract and concentrate metal cations and ligands; however, empirical data was only provided for a composite made with a single protein, bovine serum albumin (BSA).^21^ Subsequently, we have observed that the kinetics of the biomimetic mineralisation process are protein dependent. Under identical reaction conditions the precipitation of the biocomposite varies from seconds to hours and in some cases no composite is formed. For example, whilst BSA induces the formation of ZIF-8 within seconds, in our hands, a thorough study employing haemoglobin showed that aqueous solutions only yield a low quantity of non-ZIF-8 precipitate after several hours and that the precipitate does not contain protein. This observation suggests that the surface chemistry of the protein may have a significant effect on MOF crystallisation. Moreover, FTIR studies performed on proteins encapsulated within ZIF-8 point towards the existence of interactions between Zn cations and carbonyl moieties at the protein surface.^16^ To enhance our understanding of the biomimetic mineralisation process we carried out a combined computational and experimental study to investigate the role that protein surface chemistry plays in the formation of the MOF-based biocomposites. Specifically, we chemically modified the surface amino acid residues of a variety of proteins using succinic (or acetic) anhydride or ethylene diamine (Scheme S1†). Analysis of these data indicates that converting the basic residues on the protein surface into acidic or non-ionisable moieties is a convenient strategy for facilitating the biomimetic mineralisation of proteins under standard conditions.

## Results and discussion

To determine the main features of the protein chemistry that induce ZIF-8 encapsulation, we screened a series of structurally distinct proteins under identical biomimetic mineralisation conditions (0.5 mg mL^–1^ of protein dissolved in a solution composed of a 1 : 4 : 278 molar ratio of Zn^2+^ : mIM : H_2_O). These standard conditions were chosen because: (1) they have previously been shown to give rise to rapid (within seconds) biomimetic mineralisation;^21^ (2) all proteins investigated are homogeneously dispersed; and (3) a visually observable ZIF-8 precipitate is not formed in the absence of a biomacromolecule for several hours. Table 1 lists the proteins assessed for their capacity to induce the formation of a MOF-based biocomposite. Analysis of the data indicates that a biomimetically mineralised ZIF-8 precipitate is formed with proteins that have a low isoelectric point (pI) (see Fig. S1 and S2†). These proteins contain a greater proportion of acidic residues (aspartate, p*K*_a_ 3.7, and glutamate, p*K*_a_ 4.3) which will be deprotonated, and thus negatively charged under the basic reaction process.^29^ The proteins that did not induce ZIF-8 formation are those with higher pI values (above *ca.* 7) which conversely possess a larger percentage of basic amino acids (lysine, p*K*_a_ 10.5, and arginine, p*K*_a_ 12.5).^29^ We posit that basic amino acids will contribute to a positively charged protein surface, under the standard reaction conditions, and thus disfavour the accumulation of Zn^2+^ ions that engenders biomimetic mineralisation.

**Table 1:** Reported pI (pH at which the protein is uncharged), experimental zeta potential in a mIM solution at pH 11, and binary ZIF-8 growth result for each protein tested in this work. The yes/no descriptor for ZIF growth indicates the formation of a biocomposite with sodalite topology (determined by PXRD). Uncertainties are twice the standard error in the mean

Protein	pI	Ref	Zeta potential [mV]	ZIF-8	Modification	Zeta potential [mV]	ZIF-8
Pepsin	2.9	30	–30.9 ± 1.4	Yes	Amination	–7.9 ± 0.6	No
BSA	5.3	31	–36.4 ± 1.4	Yes	Amination	–5.8 ± 0.2	No
Lipase	4–8^*a*^	32	–31.7 ± 0.3	Yes			
Catalase	5.4^*b*^	33	–30.4 ± 0.6	Yes			
HRP	3.0–9.0^*c*^	34	–36.4 ± 1.0	Yes			
Haemoglobin	8.1(α), 7.0(β)	30	–21.0 ± 2.4	No	Succinylation	–37.0 ± 2.7	Yes
					Acetylation	–35.9 ± 2.6	Yes^*d*^
Myoglobin	7.6	30	–14.7 ± 2.0	No	Succinylation	–36.6 ± 0.2	Yes
					Acetylation	–36.1 ± 3.6	Yes^*d*^
Trypsin	10.7	30	–9.0 ± 1.05	No			
Lysozyme	11, 11.3	30	+6.6 ± 0.2	No			

^*a*^Broad experimental isoelectric region.

^*b*^Computational value.

^*c*^Seven isozymes.

^*d*^Not phase pure.

**Fig. 1:**
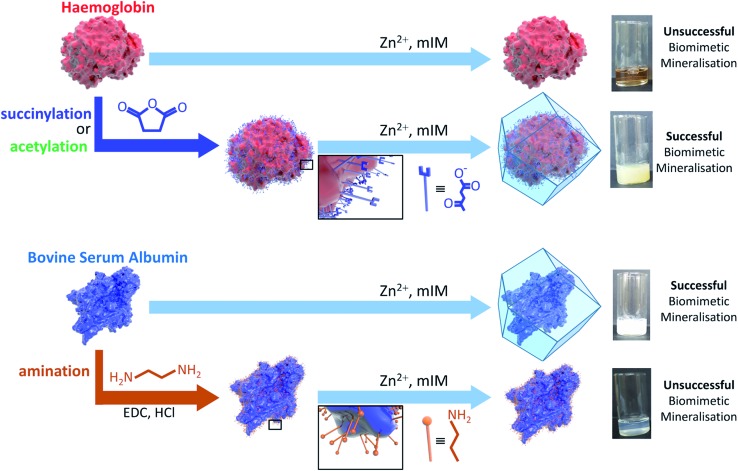
Schematic representations of the outcomes of biomimetic mineralisation for two proteins, namely haemoglobin (Hb) and bovine serum albumin (BSA). Hb does not undergo biomimetic mineralisation under standard conditions but can be chemically modified by acetylation or succinylation (shown) to increase the surface negative charge and facilitate ZIF-8 formation and encapsulation. BSA can be biomimetically mineralised but amination introduces surface amine groups that are protonated under the conditions used for ZIF-8 formation and thereby prevent mineralisation.

Amino acid modifications are commonly applied to increase the binding affinity of biomacromolecules for an immobilisation support by controlling the electrostatic interactions.^24^ Thus, we proposed this technique could be applied as a general strategy for facilitating biomimetic mineralisation under mild, standard conditions. To explore this hypothesis, we chemically modified the basic surface amino acid residues of haemoglobin (Hb) and myoglobin (Mb) that would contribute to a positive surface charge. Surface lysine residues of Hb and Mb were reacted with succinic or acetic anhydride to convert these exposed basic residues into acidic or non-ionisable groups respectively (Fig. 1 and Scheme S1†). The succinylated forms of Hb and Mb induced immediate precipitate formation upon precursor mixing. The precipitate was confirmed to have sodalite topology and rhombic dodecahedral crystal morphology characteristic of ZIF-8 by PXRD and SEM, respectively (Fig. S3–S5†). The acetylated variants, which do not provide carboxyl functional groups also facilitated precipitation of a crystalline product (Fig. S1†), but PXRD data indicated that the samples were not phase pure (Fig. S4†). These results confirm that the biomineralisation process is highly dependent on a protein's surface chemistry, with the ionisable carboxyl groups being more effective at facilitating biomineralisation of the desired ZIF-8 phase. Fig. S6† shows the UV-visible spectra of the supernatents obtained after centrifugation of the Hb and Mb biocomposites. The presence of the Soret band at 405 nm, indicates that the unmodified proteins remain in solution. To evaluate that the modified forms of these proteins were incorporated into the ZIF crystals, we performed UV-vis spectroscopy on dissolved samples of the biocomposite. We first washed the composites with SDS to ensure that surface bound protein was removed.^28^ Fig. S7† shows the UV-vis spectra of the dissolved HbAc/Succ@ZIF-8 and MbAc/Succ@ZIF-8 biocomposites. The presence of the Soret band at 405 nm is evidence that the Hb and Mb proteins are encapsulated within the ZIF-8 crystals.

Both the pI values and surface modification experiments suggest that the biomimetic mineralisation of ZIF-8 depends on electrostatics of the protein surface. Thus, we measured the zeta potential of each protein in a 160 mM mIM precursor solution to estimate their charge under the reaction conditions. The zeta potential data presented in Table 1 indicates that precipitation of ZIF-8 crystals is induced when the values are below *ca.* –30 mV. This trend explains why surface modification can switch the biomimetic mineralisation process ‘on’ or ‘off’. For example, the zeta potentials of both Hb and Mb decrease from –21 and –15 mV respectively to values significantly below –30 mV upon succinylation or acetylation. To further demonstrate the importance of surface charge, BSA and pepsin were reacted with ethylene diamine to yield a more positively charged protein. Amination of the acidic residues was confirmed by a positive shift in the zeta potential measurements above this –30 mV threshold. Both modified proteins yielded minimal precipitate, insufficient for PXRD, demonstrating an inhibition of the biomimetic mineralisation process (Fig. S2†).

The experimental data thus far confirm that the surface electrostatic potential of the biomacromolecule, which is related to, and for typical surfaces approximately equal to, the zeta potential,^35^ can be used to predict whether ZIF-8 crystallisation will be induced. These findings are consistent with previous reports that hypothesised that biomacromolecules concentrate positively charged zinc ions at their surface.^21^ Since the ion concentration varies approximately exponentially with the surface potential for purely electrostatic ion–surface interactions according to the Boltzmann equation (see Computational methods section), the surface zinc ion concentration is expected to double with each 9 mV decrease in the surface potential. This would result in an enhancement of the rate of encounters of zinc ions and mIM bridging units near the protein surface and thus to more rapid ZIF-8 formation.^36^

Both the surface electrostatic potential (zeta potential) and pI of a protein, both of which we have shown to be good discriminators of a protein's ability to seed ZIF-8 formation, can be determined from theory.^37^–^39^ Therefore, whether a protein is likely to undergo biomimetic mineralisation can be predicted prior to experimental study.

**Fig. 2:**
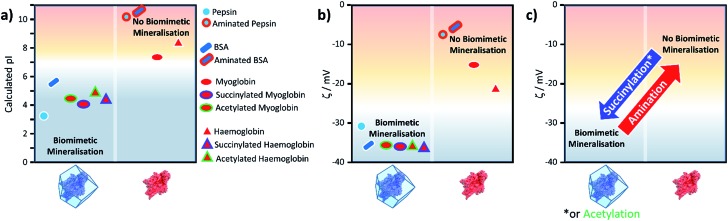
Plots of (a) the calculated pI for BSA, pepsin, Hb and Mb, with and without the surface modifications used in the experiments; (b) the experimental zeta (*ζ*) potentials for the same biomacromolecules and their modified variants; and (c) the general changes in zeta potential for the three types of chemical modifications used.

From the peptide sequence and acid-base equilibria we calculated the pI for all the proteins studied and reproduce the trend in the experimental results shown in Table 1 (Fig. 2, S9†). Furthermore, we have used the same method for computing a protein's pI to predict the effect of surface modification on propensity for ZIF-8 formation (Fig. 2, S9†).

Fig. 2 shows the calculated pI for BSA, pepsin, Hb and Mb with and without the surface modifications used in the experiments. We assumed that any target residue (lysine for acetylation and succinylation, and glutamic acid and aspartic acid for the amination) will undergo the modification reaction. As the reaction efficiency may not be 100% and our method does not consider whether amino acids are exposed to solvent, the calculated change in the pI is expected to be an overestimate; however, we get reasonable agreement to experimental values (for example aminated BSA has a pI > 9.5).^40^ As shown in Fig. 2, the calculated pI values (Fig. 2a) show the same trend as the experimental zeta potential (Fig. 2b) and clearly predicts the effect of surface modification on ZIF-8 formation for the proteins considered.

**Fig. 3:**
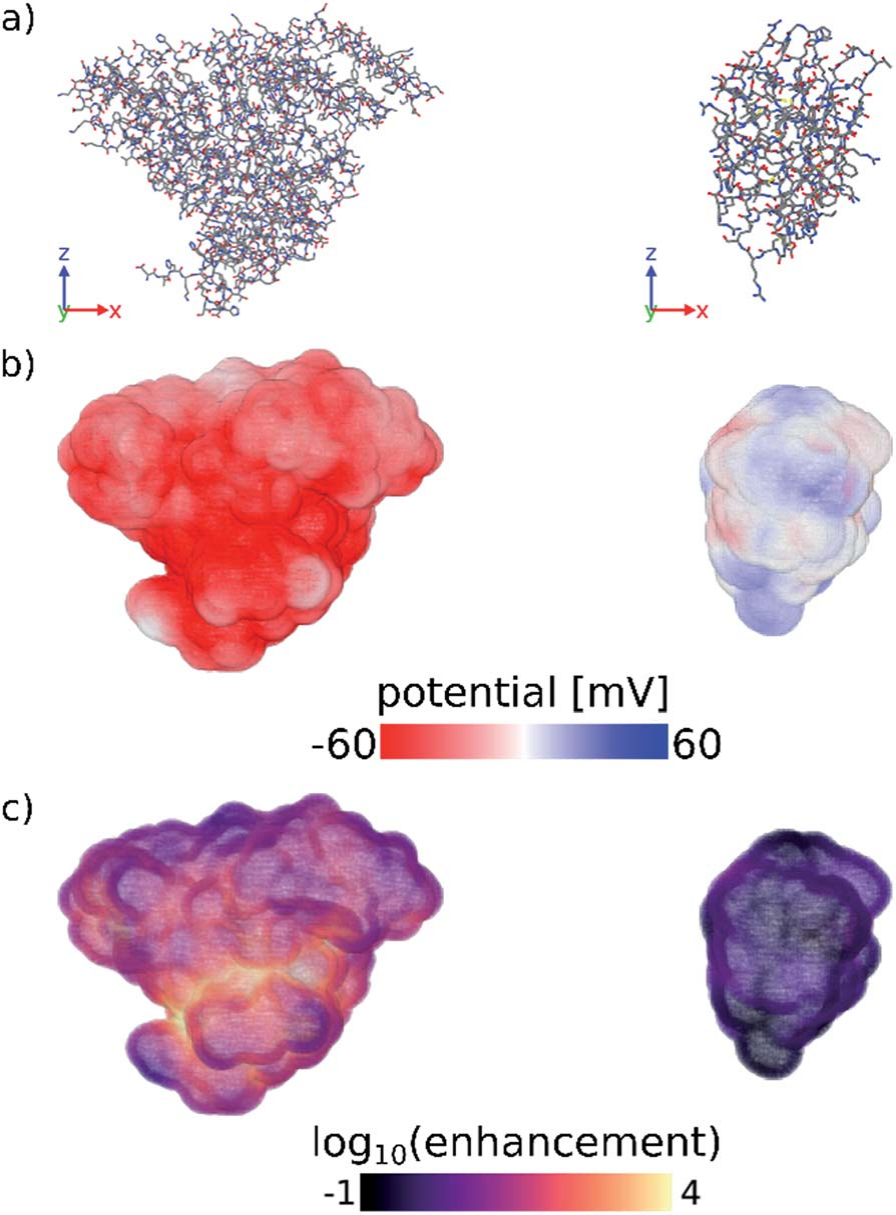
(a) Stick representations of protein crystal structures of (left) BSA and (right) lysozyme. Hydrogens are omitted for clarity. (b) Surface potential and (c) log_10_ of the zinc ion enhancement at the surface of both proteins. Zinc ion enhancement is defined as the ratio of the calculated zinc ion concentration due to the electrostatic potential and the bulk zinc ion concentration (0.04 M) at each point near the surface of the protein. Fig. S13† shows the calculated electrostatic surface of all proteins tested in this work. Figures were made using OVITO.^42^

Finally, we have computed the electrostatic potential around each of the proteins studied experimentally by solving the Poisson–Boltzmann equation, from which we can approximate the protein zeta potential (see Computational methods section and Fig. S11–S13†).^37^,^41^ The calculated surface potential also provides comprehensive 3D information about the electrostatic interactions of the protein with the surrounding electrolyte solution. Fig. 3 highlights the differences in the calculated surface potential and zinc ion enhancement at pH 11 between a protein that seeds ZIF-8 and one that does not (see also Fig. S13†). While not quantitatively reproducing the experimental data, the calculated average surface potential follows the same trend as the experimental zeta potential at pH 7 and pH 11 for the proteins studied (Fig. S11†). Importantly, we show that the predictions made by a simple sequence-based model (pI calculations) and a more physical 3D structure-based model (surface potential calculations) are equivalent, and that both of these calculations agree with our experimental observations. Combined, this supports the idea that computational screening can obviate the need for more time-consuming experimental studies.

## Conclusions

In conclusion, we have shown that the electrostatic properties of a protein's surface, as described by its pI and zeta potential, are a good predictor of whether a protein will induce ZIF-8 growth from aqueous solution. Our findings explain why the biomimetic mineralisation of ZIF-8 is not observed under standard conditions for a variety of proteins and confirm the role of Zn^2+^ concentration in seeding crystallisation. These results are consistent with studies that describe the effect of metal ion concentration gradients on the nucleation and growth of ZIF crystals.^43^ In addition, we have shown that simple chemical modification of surface ionizable residues is a convenient strategy for controlling the electrostatic potential of a protein and thus the formation of ZIF-8 biocomposites. We posit that chemical surface modification is a general strategy that can be applied to facilitate biomimetic mineralisation in a broad range of systems, including proteins, viruses and cells. Thus, this work significantly broadens the research scope and potential applications of this technique.

## Experimental

### Materials

All proteins were purchased from Sigma-Aldrich unless otherwise stated (Table S1†). Each of the proteins tested were lyophilised powders and were used without further purification. 2-Methyl imidazole (mIM) and *N*-(3-dimethylaminopropyl)-*N*′-ethylcarbodiimide hydrochloride (EDC·HCl) were purchased from Sigma-Aldrich, zinc acetate dihydrate from VWR Chemicals, succinic anhydride from BDH, acetic anhydride from Chem Supply, and ethylene diamine (EDA) from Merck. The water used was ultra pure Milli-Q (MQ) with resistivity of 18 MΩ cm^–1^ (Merck Millipore purification system). All other buffers and solvents were purchased from commercial sources and used without further purification.

### ZIF synthesis

Zn(OAc)_2_ (40 mM, 2 mL) was mixed with a solution of mIM (160 mM, 2 mL) containing the protein (2 mg). The reaction mixture was left for 16 hours undisturbed, and collected by centrifugation at 4000 rpm. The pellet was washed with water twice, followed by ethanol and air dried at ambient temperature and pressure.

### Succinylation and acetylation reactions

The method for the succinylation and acetylation of proteins was adapted from literature procedures.^44^–^46^ The protein (20 mg, haemoglobin or myoglobin) was dissolved in 4 mL of phosphate buffered saline (PBS, 100 mM, pH 8). A 50 fold molar excess of succinic anhydride or acetic anhydride was added in small increments over 1 hour. The pH was adjusted back to 8 using 2 M NaOH after each addition and the solution was stirred for 1 hour after the final addition. The protein solution was washed by ultra-filtration once with PBS (100 mM, pH 7.4) and twice with MQ water to remove excess salts (Vivacell 100, Sartorius Stedim, 10 kDa at 4000 rpm/1699 g). The protein solution was concentrated to 4 mg mL^–1^ in MQ water.

### Amination reaction

The method for the chemical amination of proteins was adapted from a literature procedure.^47^ A 2 mL solution of EDA (0.268 mL, 4.01 mmol) dissolved in MQ water was prepared and the pH was adjusted to 4.5 using 6 M HCl. The protein (20 mg, BSA or pepsin) was dissolved in the EDA solution followed by EDC·HCl (7.2 mg, 0.038 mmol). The solution was stirred on ice for 120 minutes before being washed and concentrated as described above.

### Characterisation

Powder X-ray diffraction (PXRD) data were collected on a Bruker D8-Advanced X-ray powder diffractometer (parallel X-ray, capillary-loaded) using a Cu Kα (*λ* = 1.5418 Å) radiation source. Samples were mounted in 0.5 mm glass capillaries and data collected for between 2*θ* of 2° to 52.94° with Phi rotation at 20 rotations per min at 1 second exposure per step at 5001 steps. The data were then converted into xye format and background-subtracted using WinPlotr 2000 software.^48^ Simulated powder X-ray diffraction patterns were generated from the single crystal X-ray data using Mercury 3.9.^49^

Scanning electron microscopy (SEM) images were collected using a Philips XL30 field emission scanning electron microscope (FESEM). Samples were dry loaded onto an adhesive carbon tab and sputter coated with 5 nm platinum thin film.

UV/Visible (UV/Vis) spectra were recorded at 30 °C on an Agilent Cary 60 UV/Vis spectrophotometer. Samples were diluted to 4 mL prior to each measurement.

Zeta potential measurements were recorded on a Malvern Zetasizer nano using a disposable folded cell capillary (DTS1070). Protein samples were dissolved in a HmIM solution (160 mM, pH 11) or MQ water (≈pH 7) with measurements recorded with the following parameters; Dispersant RI: 1.33, viscosity (*C*_p_): 0.887, Dispersant dielectric constant 78.5, f(*K*_a_): 1.5 (Smoluchowski approximation).

## Computational methods

### Calculation of the pI from protein sequence

For each protein, the sequence of natural amino acids was extracted from the FASTA file associated with each PDB entry (Table S1†). Using the Biopython module^50^ and the Henderson–Hasselbach equation, the average charge,1
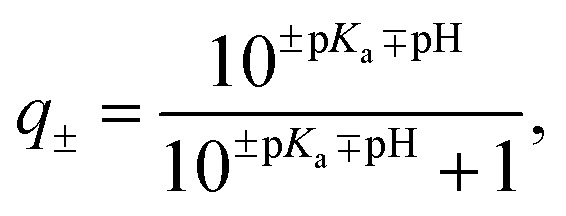
of each ionisable residue as a function of pH was calculated. The total protein charge was calculated as the sum of the average charges of all ionisable residues and the pH varied until the total protein charge was 0 ± 0.0001*e* to determine the sequence pI. The p*K*_a_ of all residue types were kept constant and defined within Biopython.

### Surface modification of proteins

The pI of surface-modified proteins was calculated using Biopython and assuming 100% efficiency of modification reactions on all target residues. For the amination reaction, any aspartate or glutamate residues were treated as lysine residues with respect to their charge and p*K*_a_. For the acetylation and succinylation reactions, any lysine residues were either ignored in the calculation of the protein charge (acetylation) or treated as glutamate residues with respect to their charge and p*K*_a_ (succinylation). See Fig. S1† for the reaction schemes.

### Calculation of average surface potentials

Crystal structures were obtained from the Protein Data Bank^51^ for each protein (PDB accession codes given in Table S1†). PROPKA 3.0 (ref. 52 and 53) was used to assign charge states to each ionisable residue in the PDB file and the PDB2PQR software^54^,^55^ was used to prepare the protein structures for analysis. See ESI† for details.

Using the SURFPOT module^37^ within the DELPHI software^41^ the linearised Poisson–Boltzmann equation,^56^

2 ∇ · [*ε*(*r*)∇*ψ*(*r*)] – *ε*_0_*ε*_r_*κ*(*r*)^2^*ψ*(*r*) = –*ρ*(*r*),

was solved to calculate the electrostatic potential, *ψ*(*r*), at position *r*. In the expression, *ρ*(*r*) is the (fixed) charge density of the solute (protein), *ε*(*r*) is the spatially varying dielectric permittivity, which is different in the protein and in the solution, and *κ*(*r*) is the Debye screening parameter given by3
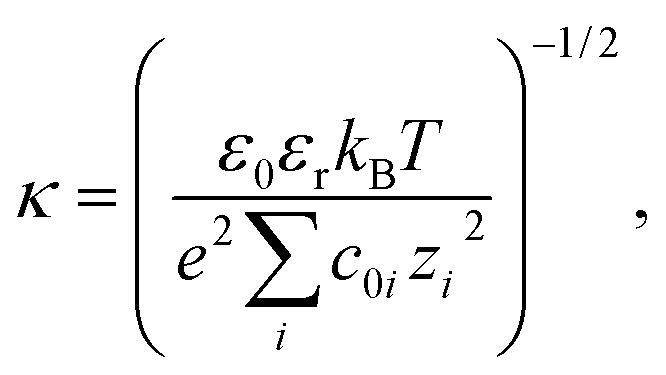
outside of the protein and is zero inside of the protein. In expression (3), *e* is the elementary charge, *k*_B_ is the Boltzmann constant, *T* is temperature, *ε*_0_ is the vacuum permittivity, *ε*_r_ is the relative permittivity of water (80), and *c*_0*i*_ and *z*_*i*_ are the bulk concentration and valency of ions of type *i*, respectively. For all our calculations the Debye length (*κ*^–1^) was 8.86 Å. The efficiency of the linearised Poisson–Boltzmann equation makes it more amenable to high-throughput computational screening than solving the full nonlinear equation and comparison of the calculated average surface potentials with experimental zeta potentials at pH 7 and pH 11 (see Fig. S11†) suggest that the linearised equation is sufficiently accurate for our purposes.

The zeta potential for each protein was estimated to be the average electrostatic potential on a surface at 4 Å from the van der Waals surface of the protein. The zeta potential of a particle undergoing electrophoresis is defined by the electrostatic potential at the shear plane, which is not readily determined for heterogeneous and rough surfaces such as proteins. The chosen surface at which the zeta potential was calculated is expected to be a reasonable approximation for the shear plane and is similar to that used previously in the literature to estimate the zeta potential of proteins.^37^ An interior protein dielectric coefficient of 4 was used and it was confirmed that the average surface potential was not sensitive to this parameter (results not shown), which agrees well with literature.^37^ We used a grid spacing of 0.5 Å, a probe radius (to define the protein surface) of 1.4 Å, which is equivalent to the radius of a water molecule, dipolar boundary conditions on the edge of the box, and a box size such that the longest dimension of the solute was 60% of the box size.

### Ion concentrations and enhancements

The concentration of ions of type *i* at position *r* was calculated from the electrostatic potential (*ψ*(*r*)) using the Boltzmann equation,4
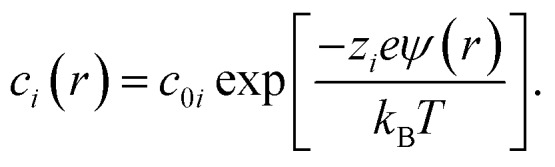



To match the experimental conditions, the bulk concentrations of the cations (zinc) and anions (acetate) in solution were taken to be *c*_0+_ = 0.04 M and *c*_0–_ = 0.08 M, respectively, and were assumed to be independent of pH. The cation and anion valencies were *z*_+_ = +2 and *z*_–_ = –1, respectively. The zinc ion enhancement (*X*(*r*)) was calculated from the ion concentration as 
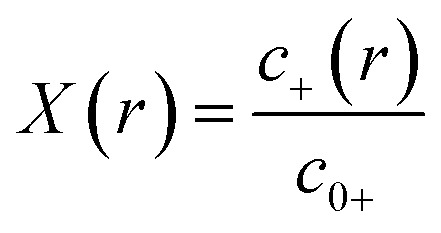
.

## Conflicts of interest

The Authors confirm that there are no conflicts to declare.

## Supplementary Material

Supplementary information
