# Supplementary material for: Protein surface functionalisation as a general strategy for facilitating biomimetic mineralisation of ZIF-8
Source: Chem Sci. 2018 Mar 9;9(18):4217–23. doi: 10.1039/c8sc00825f (PMC5942038; doi:10.1039/c8sc00825f)
Supplement: Supplementary file 1 [file SC-009-C8SC00825F-s001.pdf]

Electronic Supplementary Material (ESI) for Chemical Science.
This journal is © The Royal Society of Chemistry 2018

Supporting information for:

Protein surface functionalisation as a general strategy for facilitating biomimetic mineralisation of ZIF-8

Natasha K. Maddigan[a], Andrew Tarzia[a], David M. Huang[a], Christopher J. Sumby[a], Stephen G. Bell[a]*, Paolo Falcaro[ab]* and Christian. J. Doonan[a]*

[a.] *Department of Chemistry and the Centre for Advanced Nanomaterials, The University of Adelaide, Adelaide, South Australia 5005, Australia. Email: christian.doonan@adelaide.edu.au.*

[b.] *Institute of Physical and Theoretical Chemistry, Graz University of Technology, Stremayrgasse 9, Graz 8010, Austria.*

Contents

1. Materials 3
2. Protein surface modification reactions 3
3. Time course biomimetic mineralisation studies 4
4. Powder X-ray diffraction (PXRD) data 6
5. Scanning electron microscopy 8
6. UV-visible spectra 9
7. Additional computational methods 10
8. Additional computational results 11
9. References 16

1. Materials

Table S1. Details of the proteins, their sources, product codes and the Protein Data Bank (PDB) codes for the proteins investigated in this research.

**Protein**	**Source**	**Product Code**	**PDB File Used**[a]
Pepsin	Porcine gastric mucosa	P6887	4pep[1]
Bovine serum albumin (BSA)	Bovine	A9418	4f5s[2]
**Lipase, *Candida antarctica* Lipase B (CALB)**	*Aspergillus oryzae*	62288	1tca[3]
Catalase	Bovine Liver	C9322	3re8[4]
Peroxidase from horseradish (HRP)	Horseradish	77332	1w4w[5]
Myoglobin	Equine skeletal muscle	M0630	2frf[6]
Haemoglobin	Human	H7379	2dn2[7]
Trypsin	Porcine pancreas	T4799	1s81[8]
Lysozyme	Egg white	Astral Scientific (LDB0308)	2vb1[9]

[a] Protein structures are from the same organism from which the protein sample is sourced.

2. Protein surface modification reactions

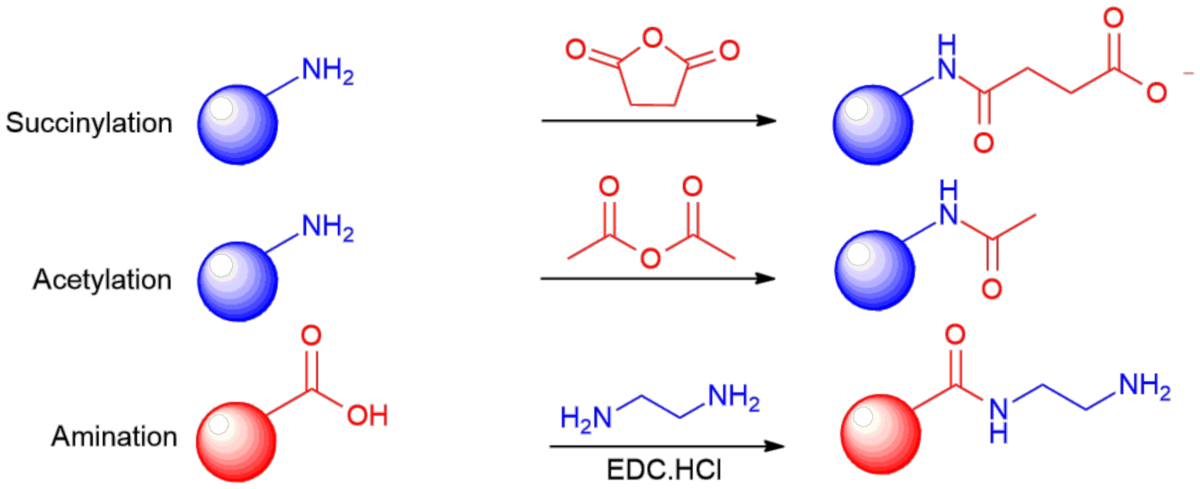

Scheme S1. Surface modification reactions. Succinylation and acetylation reactions lower the pI of a protein by modification of exposed amine groups. Amination reactions cap carboxyl groups with a free amine, thus increasing the pI.

3. Time course biomimetic mineralisation studies

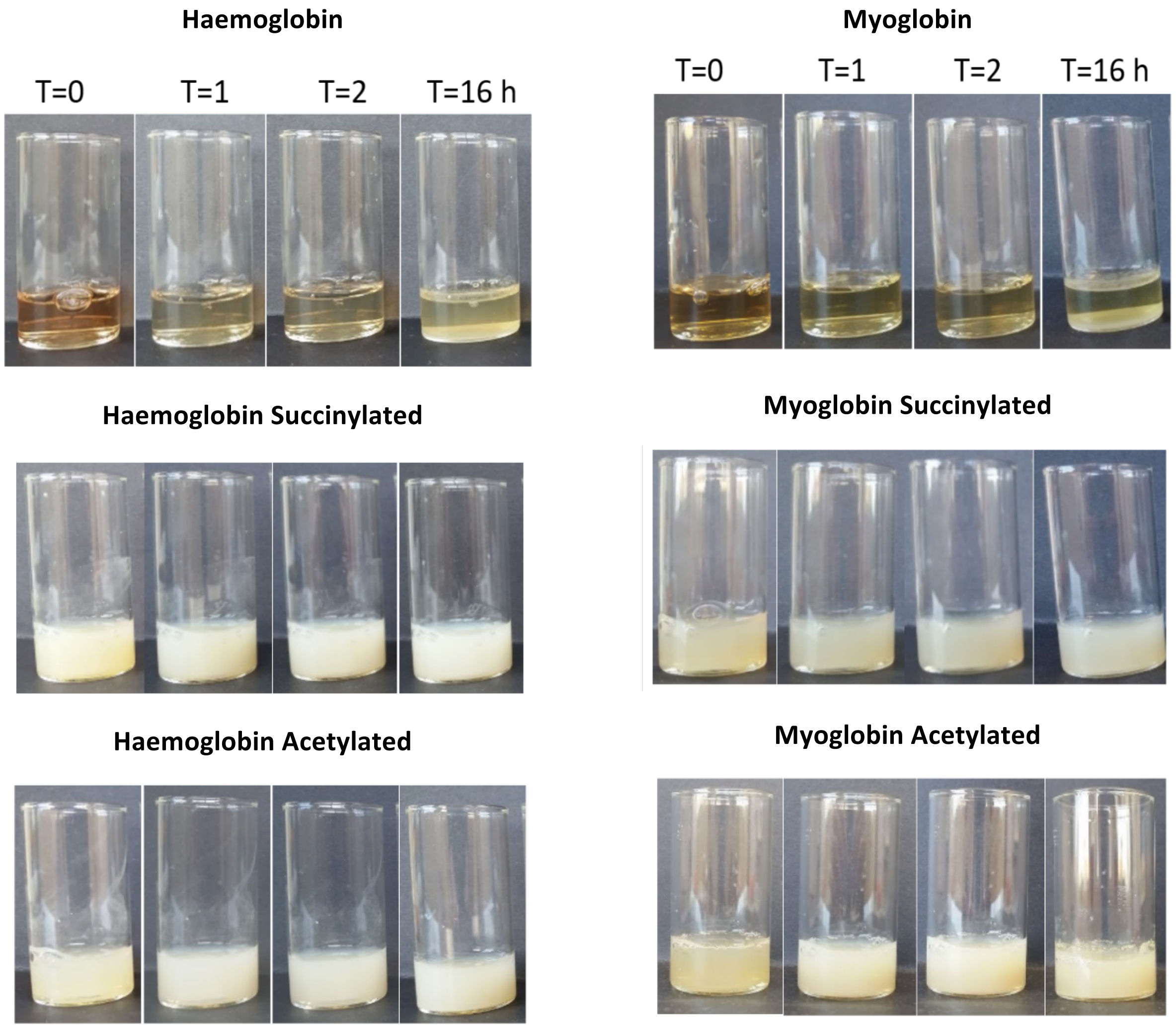

Figure S1. Sequential photographs of Haemoglobin (Hb), succinylated haemoglobin (HbSucc), acetylated haemoglobin (HbAc), myoglobin (Mb), succinylated myoglobin (MbSucc), and acetylated myoglobin (MbAc) samples (2 mg protein) immediately after mixing of the mIM (160 mM) and zinc solutions (40 mM) (T=0) until immediately prior to centrifugation and washing (T=16 hours). The unmodified haemoglobin and myoglobin samples remain clear upon addition of the zinc solution, both yielding minimal product after 16 hours. The succinylated and acetylated forms of both proteins cause immediate precipitation of ZIF.

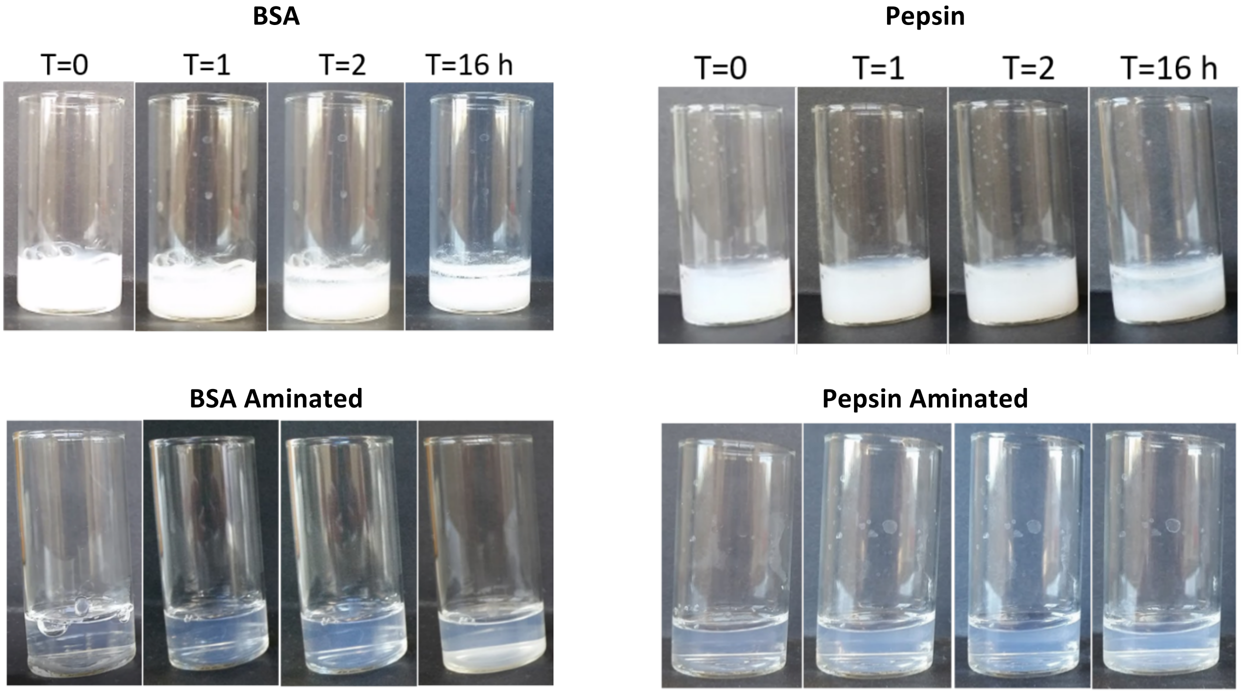

Figure S2. Sequential photographs of unmodified and aminated bovine serum albumin (BSA) and pepsin (2 mg protein) immediately after mixing of the mIM (160 mM) and zinc solutions (40 mM) (T=0) until immediately prior to centrifugation and washing (T=16 hours). The unmodified BSA and pepsin samples gave immediate biomineralization upon addition of the zinc solution. The aminated BSA and pepsin samples show a dramatic reduction in precipitation yielding only minimal product after 16 hours.

4. Powder X-ray diffraction (PXRD) data

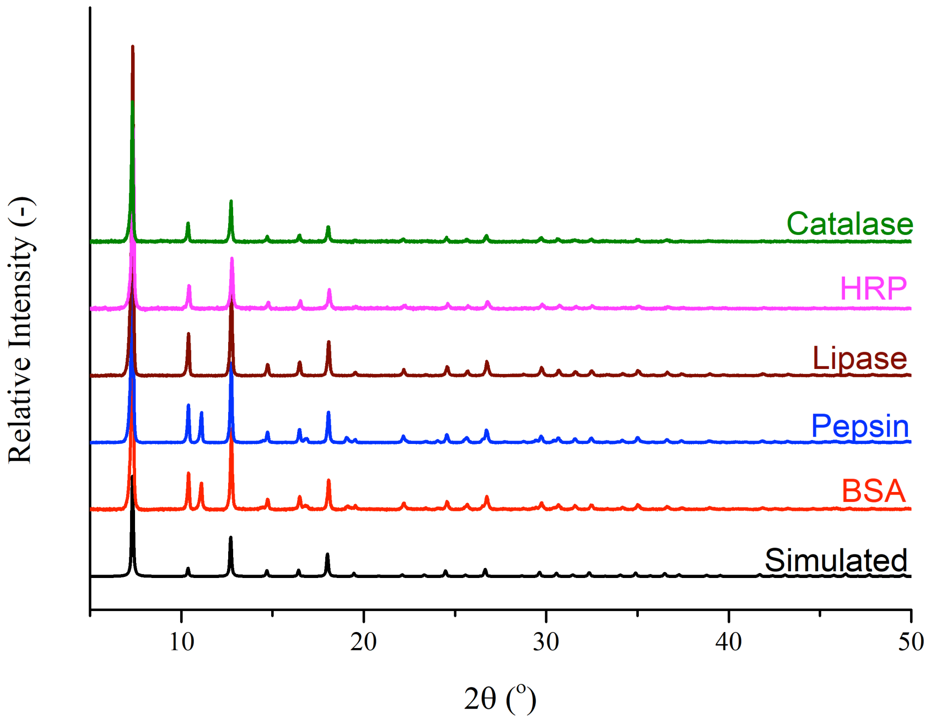

Figure S3. Powder X-ray diffraction patterns of biomimetically mineralised ZIF samples of unmodified proteins made under standard conditions (4:1 mIM:Zn^{2+}). Data collected on dried samples after washes with water and ethanol. Unmodified haemoglobin, myoglobin, lysozyme, and trypsin did not yield sufficient product for PXRD analysis. The simulated pattern relates to ZIF-8.

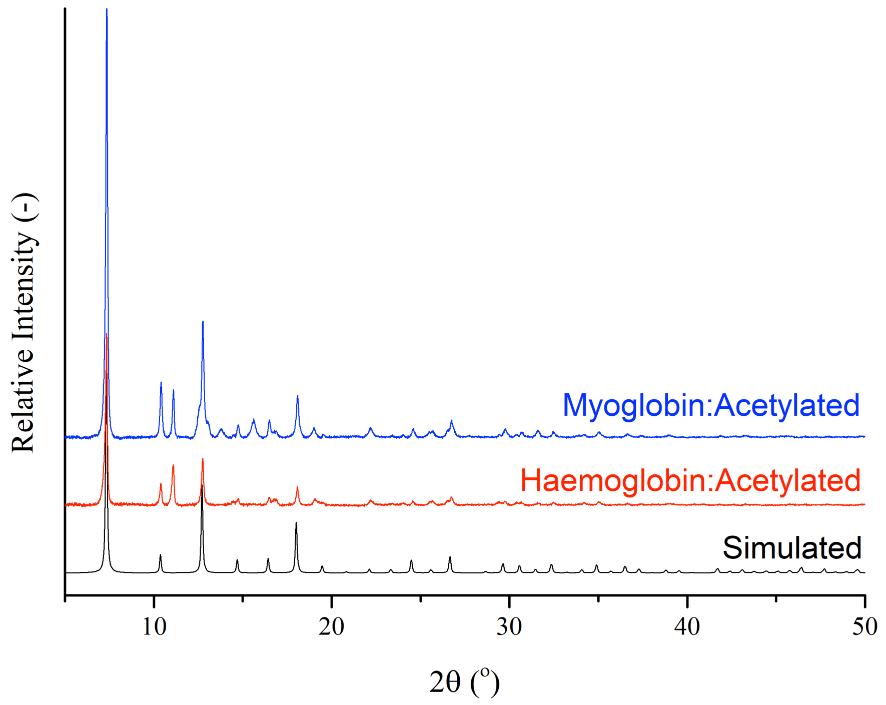

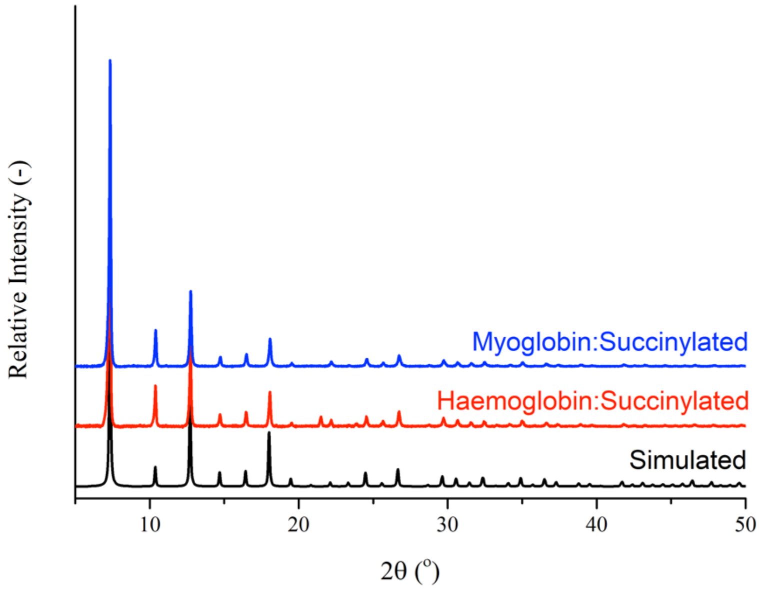

Figure S4. Powder X-ray diffraction patterns of biomimetically mineralised ZIF samples of HbSucc and MbSucc (top) and HbAc and MbAc (bottom) made under standard conditions (4:1 mIM:Zn^{2+}). Data collected on dried samples after washes with water and ethanol. Aminated BSA and pepsin, did not yield sufficient product for PXRD analysis. The simulated pattern relates to ZIF-8.

5. Scanning electron microscopy

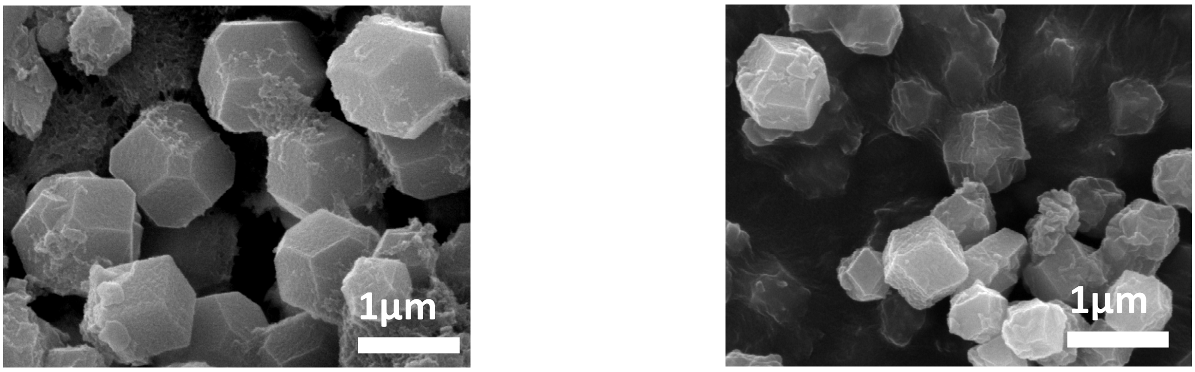

Figure S5. HbSucc@ZIF-8 (left) and MbSucc@ZIF-8 (right) after 16 hours from the beginning of the biomimetic mineralization reaction; the rhombic dodecahedral morphology can be observed.

6. UV-visible spectra

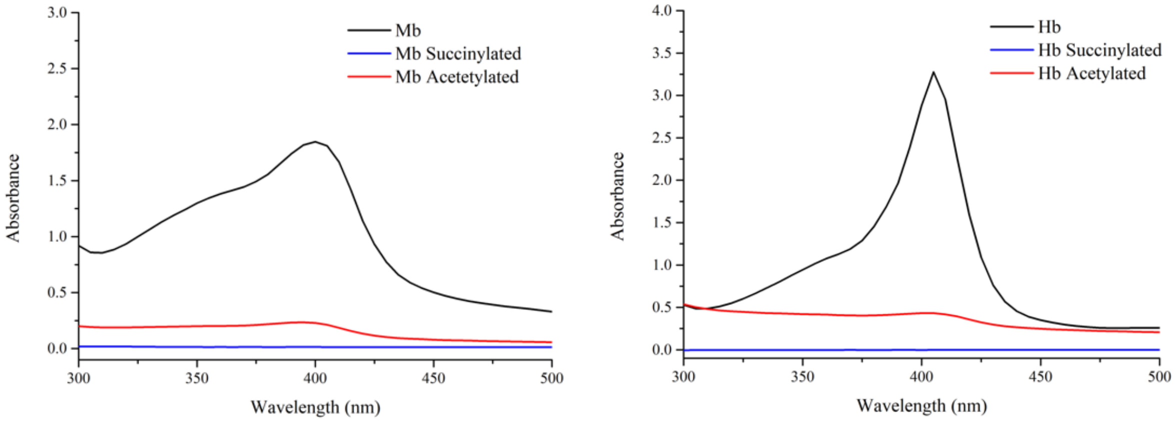

Figure S6. UV-visible spectra of the supernatant removed after centrifugation of the biocomposite samples where the protein was myoglobin (Mb), succinylated myoglobin (MbSucc), and acetylated myoglobin (MbAc) (left) and haemoglobin (Hb), succinylated haemoglobin (HbSucc), and acetylated haemoglobin (HbAc) (right). Unmodified haemoglobin and myoglobin formed minimal product and therefore show a large Soret absorbance in the removed solution, indicating that the protein has not been immobilised. In both the succinylated and acetylated variants, the absorbance has decreased thus indicating that the protein has been removed from solution and incorporated into ZIF-8 as it formed.

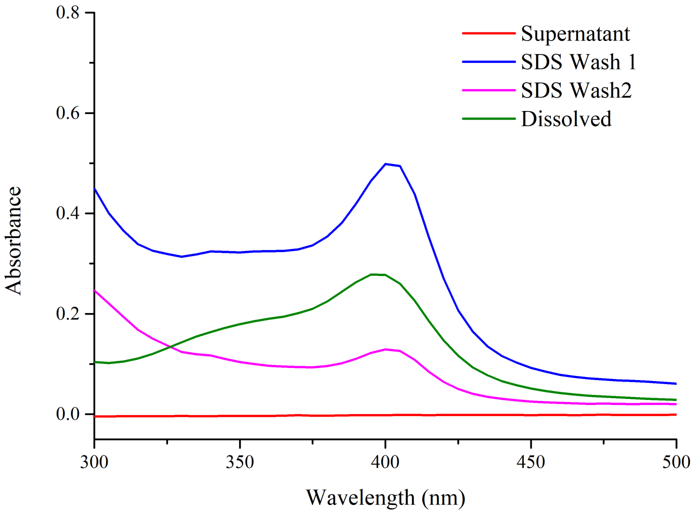

Figure S7. UV-visible spectra of the washings of succinylated haemoglobin ZIF-8 samples. The supernatant (red) was obtained after centrifugation of the product and shows no evidence of protein remaining in solution. SDS washes 1 (blue) and 2 (pink) show the appearance of the haemoglobin absorbance peak, indicating that some protein was surface bound had been washed off. After the SDS washes to remove surface bound protein showed no further protein, the ZIF-8 sample was dissolved in citric acid buffer (pH 6) containing EDTA (20 mM) and the absorbance was measured to show presence of encapsulated protein.

7. Additional computational methods

7.1. Calculation of the average hydropathic index

The hydropathic index is a measure of an amino acid sequences hydropathicity. Negative values imply an overall hydrophilic protein, whereas positive values imply an overall hydrophobic protein. The hydropathic index for a protein sequence was calculated using the Kyte and Doolittle scale of residue hydropathicity,[10] which quantifies the hydropathicity of each residue, and the Biopython module.[11, 12] A single value is reported, which is the sum of the hydropathic indices of all residues divided by the length of the sequence.

7.2. Preparation of PDB files and calculation of protein charge state

Crystal structures were obtained from the Protein Data Bank[13] for each protein (PDB accession codes given in Table S1). Where available a protein structure associated with the same organism as the experimental source was obtained. Each PDB file comes with one or more peptide chain, where each chain represents a separate sequence of amino acids in the crystal structure. For BSA, only the first polypeptide chain in the PDB file was used because this protein is expected to exist as a monomer in solution. In all other cases all chains in the PDB file were used. Heteroatoms (non-natural amino acid residues or ligands), bound ions or water molecules in the protein structures were removed.

PROPKA 3.0[14, 15] was used to estimate the pK_a of each ionisable residue in each protein structure using a highly efficient, empirical method. PROPKA uses effective potentials to calculate the total environmental perturbation to the free energy of protonation due to moving the ionisable residue from water into the 3D environment of the protein. The resultant free energy was used to determine the shift in the known pK_a of each residue due to the protein environment. We have confirmed that similar results are obtained for the calculated pK_a's using the more sophisticated DELPHIPKA[16] to assign atom charges and protonation states (results not shown). DELPHIPKA uses a variable dielectric coefficient within the protein and the free energy difference between the protonated and deprotonated state of each ionisable residue within the 3D structure (using a Poisson–Boltzmann-based approach to calculate the free energy difference) to obtain the pK_a for each residue. The calculated pK_a of each ionisable residue, given by PROPKA, was then used to calculate the 3D model pI of each protein using the Henderson–Hasselbach equation.

Before analysing each crystal structure, the PDB2PQR software[17, 18] was used to add missing heavy atoms, to make sure there were no overlapping atoms in the structure, to protonate the structure based on the pK_a's calculated by PROPKA and the given pH (where a residue is protonated if its pK_a is greater than the given pH) and to assign charges and radii from the AMBER[19] force field to each atom. We note that the AMBER force field included with PDB2PQR does not contain charge parameters for residues in certain protonation states derived by PROPKA (for example, a neutral N-terminus state is not supported by the force field provided by PDB2PQR) and therefore some residues will always exist in their pH 7 state.

8. Additional computational results

8.1. Protein metrics

In Figure S8 we show the calculated average hydropathic index of the sequence of each protein. The results indicate that the proteins that seed ZIF-8 growth have hydropathicities that overlap completely with proteins that do not seed ZIF-8 growth. This finding further supports the dominant nature of electrostatic interactions in determining ZIF-8 formation, which allows for the use of such simplified screening methods.

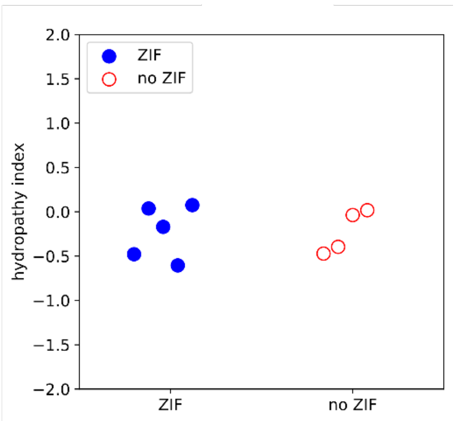

Figure S8. Categorical scatterplot of the average hydropathicity of the peptide sequences for all proteins. Closed circles are proteins that form ZIF-8 and open circles are proteins that do not form ZIF-8.

8.2. Comparison of pI from sequence models, 3D models and experiments

Figure S9 shows categorical scatter plots of the calculated pIs from the 3D structure (obtained from PROPKA 3.0) and peptide sequence (obtained from Biopython) of each protein, which shows that both calculation methods predict ZIF-8 growth reasonably accurately. Parity plots of the pIs obtained from both calculation methods as well as a comparison between the pIs calculated from the 3D protein structure and the reported pIs (Table 1) are also shown. Importantly, reasonable agreement between the two calculation methods was obtained, indicating that the much simpler sequence-based model can be used without adversely affecting prediction accuracy. We note that some experimental pIs are reported as a range of values, and so error bars are included in Figure S9d, for which the uncertainty encompasses the reported range and the pI is the mean of the reported range.

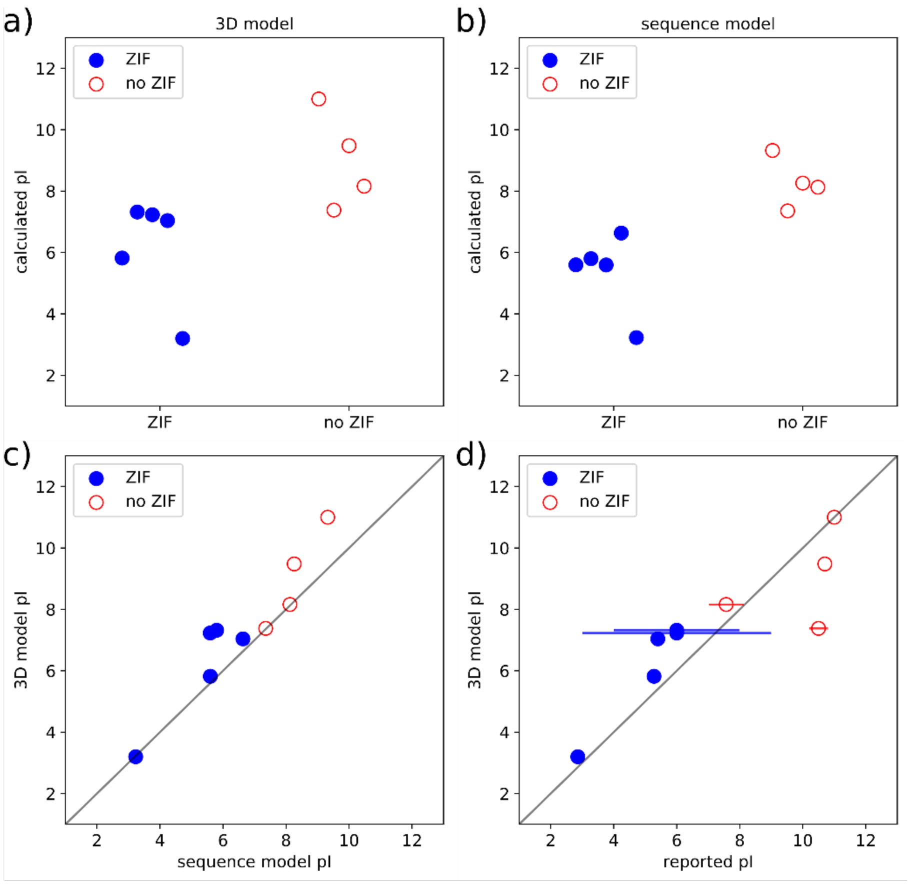

Figure S9. Categorical scatter plots of the calculated pI (a) from the 3D model and (b) sequence model of all proteins. Parity plots comparing the calculated pI from the 3D model and (c) the sequence model and (d) the reported pI values (the $y = x$ line is shown). Error bars represent ranges of pI values reported from experiments. Closed circles are proteins that form ZIF-8 and open circles are proteins that do not form ZIF-8.

8.3. Experimental zinc ion enhancement

Figure S10 shows a categorical scatter plot of the enhancement of zinc ions calculated from the experimental zeta potentials in Table 1 using Equation 4. Experimental zeta potentials give reasonable approximations of the surface electrostatic potential of each protein in solution and, therefore, a proteins ability to enhance zinc ion concentrations near the surface and, hence, seed ZIF-8 growth. Based on Figure S10, a surface zinc ion enhancement of > 10, which is a zinc ion concentration of 0.4 M, leads to ZIF-8 formation under experimental conditions.

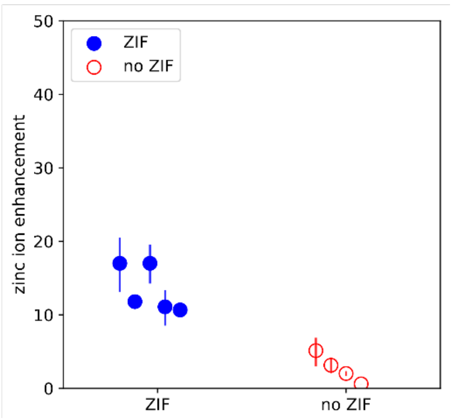

Figure S10. Categorical scatterplots of the zinc ion enhancements calculated from the experimental zeta potentials at pH 11 for all proteins. Closed circles are proteins that form ZIF-8 and open circles are proteins that do not form ZIF-8.

8.4. Zinc ion enhancement from 3D model

The calculated average surface potentials from the 3D model of each protein show reasonable agreement with the experimental zeta potentials (Figure S11). The main discrepancies are the overestimation of the average surface potential compared with experimental zeta potentials for very highly charged proteins, such as BSA, catalase and pepsin. This result is not unexpected, given the use of the linearised Poisson–Boltzmann equation, which breaks down in regimes of high zeta potential ($|\zeta| > \left|\frac{k_B T}{z_+ e}\right| \approx 12\ \mathrm{mV}$). The underestimation of the average surface potential compared with experimental zeta potentials for lipase and HRP is likely a result of experimental impurities. Both proteins are expected to be glycosylated,[20, 21] which is known to affect zeta potential measurements,[22] whereas the calculations used non-glycosylated structures. Additionally, HRP could be a mixture of different iso-enzymes with vastly different electrostatic properties.[23] We note that both proteins have reported pIs that span a broad range of values (Table 1), indicating a broad range of electrostatic properties for different samples.

Our calculation methodology used static 3D structures of each protein obtained from X-ray crystallography, which are unlikely to be representative of the protein structure in solution at a pH of 11. At high pHs, the presence of high-charge regions would lead to repulsion and a degree of unfolding, which such a simple model could not take into account. We also note that it has been shown previously that the interior of a protein has a highly variable dielectric coefficient and assuming a constant dielectric coefficient, as we have, can give rise to errors near the surface of proteins.[24] Furthermore, by taking the average surface potential to be equal to the experimental zeta potential for a heterogenous protein surface we assumed that the electric double layer surrounding the protein is thin compared with the size of the protein and that the linearised Poisson–Boltzmann equation applies, which may not always be the case for the systems studied (discussed above).[25] The semi-quantitative agreement with experiment in most cases is very encouraging, considering the approximations in the calculations.

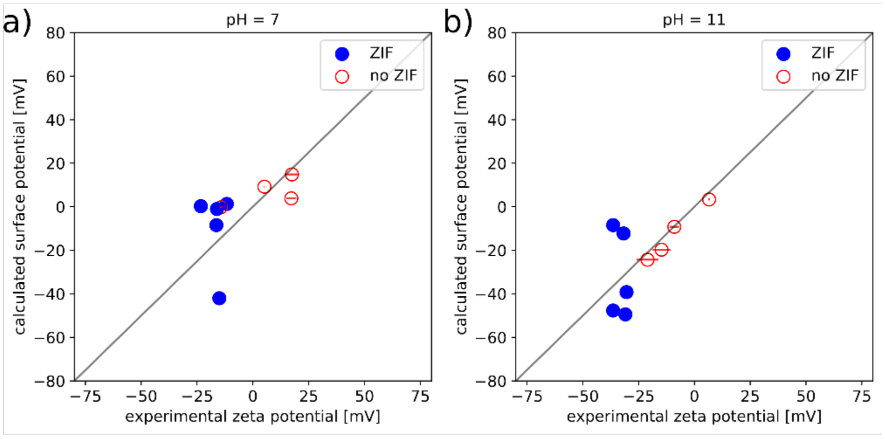

Figure S11. Parity plots comparing the calculated surface potential from our 3D model and experimental zeta potentials at (a) pH 7 and (b) pH 11 for all proteins (the $y = x$ line is shown). Closed circles are proteins that form ZIF-8 and open circles are proteins that do not form ZIF-8.

Figure S12 shows a categorical scatter plot of the calculated average surface potentials at pH 9 and pH 11 for all proteins. These results support the experimental findings and show a reasonable ability to predict a protein's propensity to seed ZIF-8 formation. Results at pH 9 and pH 11 are shown as the initial solution (before zinc ions are added) is at approximately pH 11, but upon zinc ion addition, the pH quickly decreases to around 9, likely because of ZIF nucleation.[26] Finally, Figure S13 shows the variation in the surface potential around all proteins at pH 11 calculated from our 3D model.

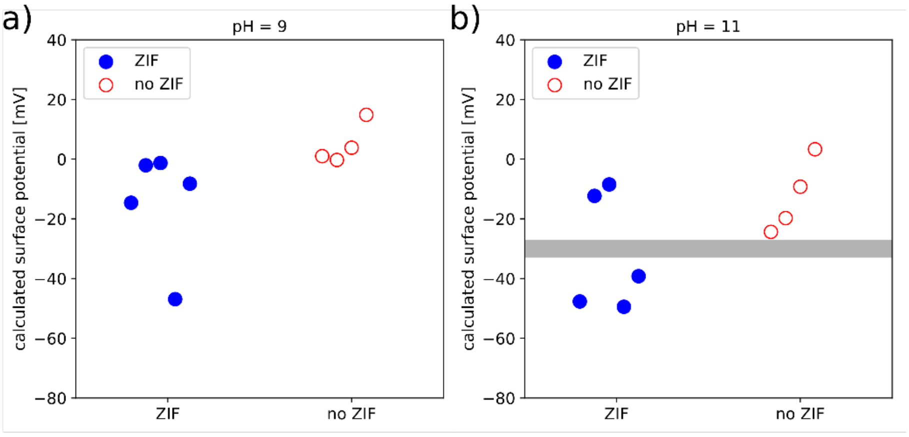

Figure S12. Categorical scatterplots of the calculated surface potential from the 3D model (a) at pH 9 and (b) pH 11 for all proteins. Closed circles are proteins that form ZIF-8 and open circles are proteins that do not form ZIF-8. The shaded region highlights the approximate boundary of the zeta potential in the experiments for proteins that do and do not seed ZIF-8 growth.

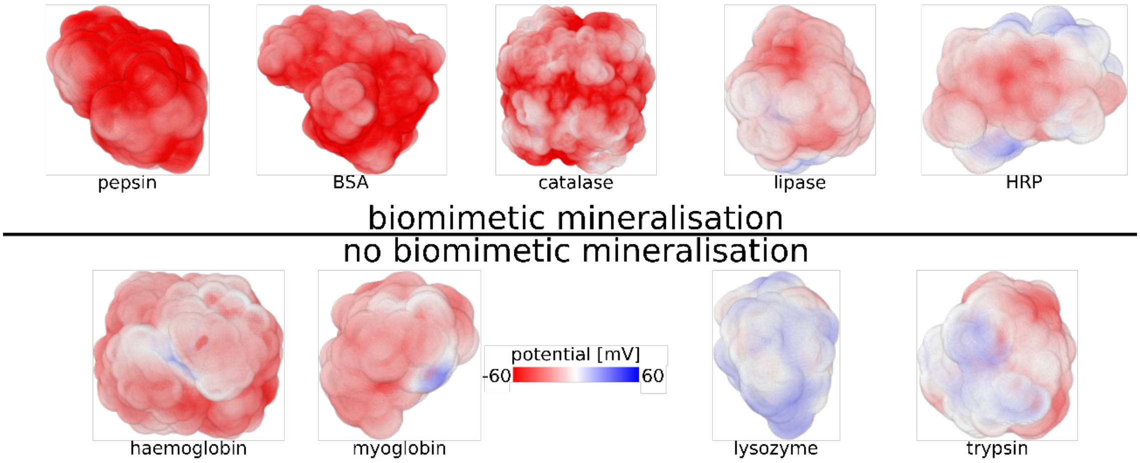

Figure S13. Surface potential surrounding all proteins calculated from our 3D model at pH 11. Lipase and HRP are outliers based on our analysis.

9. References

1. A. R. Sielecki, A. A. Fedorov, A. Boodhoo, N. S. Andreeva and M. N. G. James, *J. Mol. Biol.*, 1990, **214**, 143-170.
2. A. Bujacz, *Acta Crystallogr. D*, 2012, **68**, 1278-1289.
3. J. Uppenberg, M. T. Hansen, S. Patkar and T. A. Jones, *Structure*, 1994, **2**, 293-308.
4. N. Purwar, J. M. McGarry, J. Kostera, A. A. Pacheco and M. Schmidt, *Biochem.*, 2011, **50**, 4491-4503.
5. G. H. Carlsson, P. Nicholls, D. Svistunenko, G. I. Berglund and J. Hajdu, *Biochem.*, 2005, **44**, 635-642.
6. D. M. Copeland, A. S. Soares, A. H. West and G. B. Richter-Addo, *J. Inorg. Biochem.*, 2006, **100**, 1413-1425.
7. S.-Y. Park, T. Yokoyama, N. Shibayama, Y. Shiro and J. R. H. Tame, *J. Mol. Biol.*, 2006, **360**, 690-701.
8. T. R. Transue, J. M. Krahn, S. A. Gabel, E. F. DeRose and R. E. London, *Biochem.*, 2004, **43**, 2829-2839.
9. J. Wang, M. Dauter, R. Alkire, A. Joachimiak and Z. Dauter, *Acta Crystallogr. D*, 2007, **63**, 1254-1268.
10. J. Kyte and R. F. Doolittle, *J. Mol. Biol.*, 1982, **157**, 105-132.
11. T. Hamelryck and B. Manderick, *Bioinformatics*, 2003, **19**, 2308-2310.
12. P. J. A. Cock, T. Antao, J. T. Chang, B. A. Chapman, C. J. Cox, A. Dalke, I. Friedberg, T. Hamelryck, F. Kauff, B. Wilczynski and M. J. L. de Hoon, *Bioinformatics*, 2009, **25**, 1422-1423.
13. H. M. Berman, J. Westbrook, Z. Feng, G. Gilliland, T. N. Bhat, H. Weissig, I. N. Shindyalov and P. E. Bourne, *Nucleic Acids Res.*, 2000, **28**, 235-242.
14. C. R. Søndergaard, M. H. M. Olsson, M. Rostkowski and J. H. Jensen, *J. Chem. Theory Comput.*, 2011, **7**, 2284-2295.
15. M. H. M. Olsson, C. R. Søndergaard, M. Rostkowski and J. H. Jensen, *J. Chem. Theory Comput.*, 2011, **7**, 525-537.
16. L. Wang, L. Li and E. Alexov, *Proteins: Struct., Funct., Bioinf.*, 2015, **83**, 2186-2197.
17. T. J. Dolinsky, J. E. Nielsen, J. A. McCammon and N. A. Baker, *Nucleic Acids Res.*, 2004, **32**, W665-W667.
18. T. J. Dolinsky, P. Czodrowski, H. Li, J. E. Nielsen, J. H. Jensen, G. Klebe and N. A. Baker, *Nucleic Acids Res.*, 2007, **35**, W522-W525.
19. J. Wang, P. Cieplak and P. A. Kollman, *J. Comput. Chem.*, 2000, **21**, 1049-1074.
20. O. Spadiut, L. Rossetti, C. Dietzsch and C. Herwig, *Protein Expr. Purif.*, 2012, **86**, 89-97.
21. I. Høegh, S. Patkar, T. Halkier and M. T. Hansen, *Can. J. Bot.*, 1995, **73**, 869-875.
22. M. Kreuß, T. Strixner and U. Kulozik, *Food Hydrocolloids*, 2009, **23**, 1818-1826.
23. M. C. Hoyle, *Plant Physiol.*, 1977, **60**, 787.
24. L. Li, C. Li, Z. Zhang and E. Alexov, *J. Chem. Theory Comput.*, 2013, **9**, 2126-2136.
25. F. Fogolari, P. Zuccato, G. Esposito and P. Viglino, *Biophys. J.*, 1999, **76**, 1-16.
26. M. Jian, B. Liu, R. Liu, J. Qu, H. Wang and X. Zhang, *RSC Adv.*, 2015, **5**, 48433-48441.
